# Supplementary material for: ProteinShader: illustrative rendering of macromolecules
Source: BMC Struct Biol. 2009 Mar 30;9:19. doi: 10.1186/1472-6807-9-19 (PMC2672931; doi:10.1186/1472-6807-9-19)
Supplement: Additional file 1 — ProteinShader program without source code. This compressed file contains the complete ProteinShader program including associated libraries, but no source code. A README.txt file gives an overview of the ProteinShader distribution, and the index.html file in the help subdirectory has directions on getting started with the program as well as a set of tutorials. [file 1472-6807-9-19-S1.zip › ProteinShader-beta-0_9_4-binary/help/api/org/proteinshader/gui/listeners/menubar/FileMenuListenerFactory.html]

FileMenuListenerFactory (ProteinShader API)


|  |  |  |
| --- | --- | --- |
| **Overview** \| **Package** \| **Class** \| **Use** \| **Tree** \| **Deprecated** \| **Index** \| **Help** | |  |
| **PREV CLASS**   **NEXT CLASS** | **FRAMES**    **NO FRAMES**     **All Classes** |  |
| SUMMARY: NESTED \| FIELD \| CONSTR \| METHOD | DETAIL: FIELD \| CONSTR \| METHOD |  |


---


## org.proteinshader.gui.listeners.menubar Class FileMenuListenerFactory

```
java.lang.Object
  org.proteinshader.gui.listeners.menubar.FileMenuListenerFactory
```

---

``` public class FileMenuListenerFactory extends Object ```

Creates all event listeners needed by the FileMenu of the MainMenuBar.

---

**Constructor Summary**

`FileMenuListenerFactory(Mediator mediator)`
  
          Constructs a FileMenuListenerFactory.


| **Method Summary** | |
| --- | --- |
| `ActionListener` | `createImageFileMenuItemListener()`             Creates an action listener for the "PNG File..." menu item. |
| `ActionListener` | `createOpenMenuItemListener()`             Creates an action listener for the "Open" menu item. |
| `ActionListener` | `createQuitMenuItemListener()`             Creates an action listener for the "Quit" menu item. |

**Methods inherited from class java.lang.Object**

`clone, equals, finalize, getClass, hashCode, notify, notifyAll, toString, wait, wait, wait`

**Constructor Detail**

### FileMenuListenerFactory

```
public FileMenuListenerFactory(Mediator mediator)
```

:   Constructs a FileMenuListenerFactory.

    **Parameters:**: `mediator` - the centralized Mediator object that listeners call on to accomplish their task.


**Method Detail**

### createOpenMenuItemListener

```
public ActionListener createOpenMenuItemListener()
```

:   Creates an action listener for the "Open" menu item. When the
    action listener is called, it will obtain a filename from the user
    and then call on the Mediator to read the file and create a new
    Structure.

    :   **Returns:**: The action listener.

---


### createImageFileMenuItemListener

```
public ActionListener createImageFileMenuItemListener()
```

:   Creates an action listener for the "PNG File..." menu item. When the action
    listener is called, it will save the current canvas image to a ".png" file.
    The images will be saved in the FileMenu.IMAGES\_DIRECTORY, which will be
    created if it does not already exist.

    :   **Returns:**: The action listener.

---


### createQuitMenuItemListener

```
public ActionListener createQuitMenuItemListener()
```

:   Creates an action listener for the "Quit" menu item. When the
    action listener is called, it will terminate the program.

    :   **Returns:**: The action listener.


---


|  |  |  |
| --- | --- | --- |
| **Overview** \| **Package** \| **Class** \| **Use** \| **Tree** \| **Deprecated** \| **Index** \| **Help** | |  |
| **PREV CLASS**   **NEXT CLASS** | **FRAMES**    **NO FRAMES**     **All Classes** |  |
| SUMMARY: NESTED \| FIELD \| CONSTR \| METHOD | DETAIL: FIELD \| CONSTR \| METHOD |  |


---

# *Copyright © 2007-2008*
